# Model-Predicted Impact of ECG Monitoring Strategies During Bedaquiline Treatment

**DOI:** 10.1093/ofid/ofac372

**Published:** 2022-07-27

**Authors:** Stijn W van Beek, Lénaïg Tanneau, Graeme Meintjes, Sean Wasserman, Neel R Gandhi, Angie Campbell, Charle A Viljoen, Lubbe Wiesner, Rob E Aarnoutse, Gary Maartens, James C M Brust, Elin M Svensson

**Affiliations:** Department of Pharmacy, Radboud Institute for Health Sciences, Radboud University Medical Center, Nijmegen, the Netherlands; Department of Pharmacy, Uppsala University, Uppsala, Sweden; Department of Medicine, Wellcome Centre for Infectious Diseases Research in Africa, Institute of Infectious Disease and Molecular Medicine, University of Cape Town, Cape Town, South Africa; Department of Medicine, Wellcome Centre for Infectious Diseases Research in Africa, Institute of Infectious Disease and Molecular Medicine, University of Cape Town, Cape Town, South Africa; Division of Infectious Diseases and HIV Medicine, Department of Medicine, University of Cape Town, Cape Town, South Africa; Departments of Epidemiology & Global Health, Rollins School of Public Health, Emory University, Atlanta, Georgia, USA; Division of Infectious Diseases, Department of Medicine, Emory School of Medicine, Emory University, Atlanta, Georgia, USA; Departments of Epidemiology & Global Health, Rollins School of Public Health, Emory University, Atlanta, Georgia, USA; Division of Cardiology, Department of Medicine, University of Cape Town, Cape Town, South Africa; Cape Heart Institute, Faculty of Health Sciences, University of Cape Town, Cape Town, South Africa; Division of Clinical Pharmacology, Department of Medicine, University of Cape Town, Cape Town, South Africa; Department of Pharmacy, Radboud Institute for Health Sciences, Radboud University Medical Center, Nijmegen, the Netherlands; Department of Medicine, Wellcome Centre for Infectious Diseases Research in Africa, Institute of Infectious Disease and Molecular Medicine, University of Cape Town, Cape Town, South Africa; Division of Clinical Pharmacology, Department of Medicine, University of Cape Town, Cape Town, South Africa; Division of General Internal Medicine, Department of Medicine, Albert Einstein College of Medicine, Bronx, New York, USA; Department of Pharmacy, Radboud Institute for Health Sciences, Radboud University Medical Center, Nijmegen, the Netherlands; Department of Pharmacy, Uppsala University, Uppsala, Sweden

**Keywords:** bedaquiline, QT-interval prolongation, tuberculosis, modeling, ECG monitoring

## Abstract

**Background:**

The M2 metabolite of bedaquiline causes QT-interval prolongation, making electrocardiogram (ECG) monitoring of patients receiving bedaquiline for drug-resistant tuberculosis necessary. The objective of this study was to determine the relationship between M2 exposure and Fridericia-corrected QT (QTcF)-interval prolongation and to explore suitable ECG monitoring strategies for 6-month bedaquiline treatment.

**Methods:**

Data from the PROBeX study, a prospective observational cohort study, were used to characterize the relationship between M2 exposure and QTcF. Established nonlinear mixed-effects models were fitted to pharmacokinetic and ECG data. In a virtual patient population, QTcF values were simulated for scenarios with and without concomitant clofazimine. ECG monitoring strategies to identify patients who need to interrupt treatment (QTcF > 500 ms) were explored.

**Results:**

One hundred seventy patients were included, providing 1131 bedaquiline/M2 plasma concentrations and 1702 QTcF measurements; 2.1% of virtual patients receiving concomitant clofazimine had QTcF > 500 ms at any point during treatment (0.7% without concomitant clofazimine). With monthly monitoring, almost all patients with QTcF > 500 ms were identified by week 12; after week 12, patients were predominantly falsely identified as QTcF > 500 ms due to stochastic measurement error. Following a strategy with monitoring before treatment and at weeks 2, 4, 8, and 12 in simulations with concomitant clofazimine, 93.8% of all patients who should interrupt treatment were identified, and 26.4% of all interruptions were unnecessary (92.1% and 32.2%, respectively, without concomitant clofazimine).

**Conclusions:**

Our simulations enable an informed decision for a suitable ECG monitoring strategy by weighing the risk of missing patients with QTcF > 500 ms and that of interrupting bedaquiline treatment unnecessarily. We propose ECG monitoring before treatment and at weeks 2, 4, 8, and 12 after starting bedaquiline treatment.

Tuberculosis claims 1.5 million lives annually and was the leading cause of death from a single infectious agent until the emergence of coronavirus disease 2019 (COVID-19) in 2020 [1]. Drug-resistant tuberculosis is a particularly large threat to global public health, and only 3 new drugs have been added to the limited treatment options over the past 50 years [1]. Bedaquiline was conditionally approved by the US Food and Drug Administration (FDA) in 2012 and was the first antituberculosis drug from a novel class since the approval of rifampicin in 1971 [2]. It significantly improves treatment outcomes in drug-resistant tuberculosis, and the World Health Organization (WHO) recommends including it in treatment regimens for all patients with rifampicin-resistant tuberculosis [3].

Bedaquiline causes QT-interval prolongation, driven primarily by its M2 metabolite, and as a result, electrocardiogram (ECG) monitoring of patients receiving bedaquiline is necessary [2–5]. QT-interval prolongation increases the risk of *torsades de pointes*, an arrhythmic event that can lead to sudden death. An interval of >500 ms warrants interruption of QT-prolonging drugs. Both the labels of the FDA and the European Medicines Agency (EMA) for bedaquiline give recommendations for when to perform ECG monitoring [2, 4]. The FDA label suggests ECG monitoring before starting treatment and at least at weeks 2, 12, and 24. The EMA label states that ECG monitoring should be performed before starting treatment and at least monthly during treatment. The 2014 WHO guidelines recommended the same ECG monitoring as the FDA, but with more intensive monthly monitoring if other QT-prolonging drugs or lopinavir/ritonavir (LPVr) are co-administered [6]. However, general ECG monitoring recommendations are not included in updated WHO guidelines on tuberculosis and drug-resistant tuberculosis treatment [3, 7, 8, 9]. Furthermore, a solid evidence base informing an optimal ECG monitoring strategy is lacking.

The Pharmacokinetics, Resistance, and Outcomes of Bedaquiline in multidrug-resistant and eXtensively drug-resistant tuberculosis (PROBeX) study was a prospective observational cohort study conducted between 2016 and 2020 in South Africa [10]. The PROBeX study included 195 rifampicin-resistant tuberculosis patients who started bedaquiline treatment from 3 drug-resistant tuberculosis referral hospitals. Only 4 of the 195 patients (2%) experienced a heart rate–corrected QT interval using Fridericia’s formula (QTcF) of >500 ms. Five patients (3%) interrupted bedaquiline treatment due to QTcF prolongation, but none experienced a QTcF >500 ms, and all eventually restarted bedaquiline.

The objective of the current analysis was to determine the relationship between M2 pharmacokinetics (PK) and QTcF-interval prolongation in the PROBeX study and to explore suitable ECG monitoring strategies for bedaquiline treatment using modeling and simulation methods.

## METHODS

### Patients and Study Design

Patient characteristics, PK, QTcF, serum albumin, and electrolyte data were obtained from 195 participants in the PROBeX study. The study was approved by the institutional review boards of the involved universities, and all participants signed written informed consent. It included adult patients who were starting bedaquiline-containing treatment regimens. The standard regimen typically included bedaquiline, linezolid, clofazimine, levofloxacin, ethionamide, terizidone, and pyrazinamide. The treatment duration was 6 months for bedaquiline and up to 24 months for the regimen. The bedaquiline treatment regimen consisted of 400 mg daily for 2 weeks, followed by 200 mg 3 times weekly for 22 weeks. All participants started bedaquiline treatment after inclusion in the study, but they could have received other antituberculosis therapy before enrollment. Following the standard of care, participants receiving moxifloxacin at enrollment were switched to levofloxacin, which is known to cause less QTcF-interval prolongation. The majority (63%) of the PROBeX study participants were living with HIV. Participants with HIV received antiretroviral therapy based on either nevirapine or LPVr.

Single PK plasma samples were collected at months 1, 2, and 6 of bedaquiline treatment, aiming to sample at ∼6–12 hours after the last bedaquiline administration. Consecutive participants from a single center in Cape Town were invited to participate in a PK substudy that had additional intensive PK sampling at month 2 with plasma sampling at predose and 1, 2, 3, 4, 5, 6, and 24 hours after bedaquiline administration. Single PK samples were also collected at 3 and 6 months after finishing bedaquiline treatment as part of the PK substudy. Plasma concentrations of bedaquiline and its metabolite M2 were determined for each plasma sample using validated high-performance liquid chromatography as described previously [11]. The lower limits of quantification for bedaquiline and M2 were 20 ng/mL and 10 ng/mL, respectively. The interday accuracy of both bedaquiline and M2 ranged from 95.1% to 100.1% during sample analysis, and the imprecision from 4.2% to 7.7%.

ECG measurements were performed before starting bedaquiline treatment and at 1, 2, and 6 months after treatment start. Trained study staff performed ECGs in triplicate with at least 5 minutes between measurements. QT intervals were measured manually by a single cardiologist (C.A.V.) and were corrected for heart rate using Fridericia’s formula (QTcF = [QT interval]/[RR interval]^1/3^) [12]. The ECG measurements performed for the study were independent from the ECG measurements as part of routine monthly safety monitoring done by clinic providers. The latter ECG measurements were not included in this analysis. Although clinical service providers were notified by study staff if a study ECG showed a prolonged QTcF, they typically repeated ECGs themselves and made their treatment decisions accordingly.

### Pharmacokinetic/Pharmacodynamic Modeling

We used a previously established model to fit the plasma PK of bedaquiline and M2 with maximum a posteriori estimation (using the MAXEVAL = 0 and POSTHOC options in the NONMEM estimation step to estimate individual parameters) [13]. This model includes submodels characterizing body weight and albumin over time, as both typically increase during treatment and significantly affect the PK of bedaquiline and its M2 metabolite. The earlier model also identified age and race as significant covariates. We accounted for the inhibitory effect of LPVr treatment on the plasma clearances of bedaquiline and M2 by implementing a decrease in clearances according to previously reported values [14]. The fit of the individual model-predicted plasma concentrations to the observed concentrations was visually assessed (the goodness-of-fit plot of individual observed vs predicted M2 concentrations is included in the Supplementary Data). As plasma PK samples were not available at the exact times of all ECG measurements, model-predicted M2 concentrations were used to characterize the relationship with QTcF prolongation.

To characterize bedaquiline-induced QTcF-interval prolongation, we used the model structure from a previously established model describing the E_max_ relationship between M2 concentration and QTcF prolongation [5]. The original model’s parameter values were implemented as informative priors [15]. The QTcF model accounted for the effects of circadian rhythm, time on bedaquiline treatment, concurrent clofazimine and/or moxifloxacin use, age, sex, race, and calcium and potassium on the baseline QTcF interval. As an E_max_ model was found to better describe the relationship between M2 concentration and QTcF prolongation than a linear model during the development of the original QTcF model, we did not explore a linear model in the current analysis.

### ECG Monitoring Simulations

Patient demographics and electrolyte values in the simulations were based on those from PROBeX study participants. Individuals with a baseline QTcF >450 ms were excluded from participating in the PROBeX study, and the typical baseline QTcF we estimate may not be representative for the general population. To compensate for this, we increased the typical baseline QTcF and doubled its interindividual variability (IIV). Based on a South African retrospective cohort study of 420 patients receiving bedaquiline treatment, the typical baseline QTcF was increased from 400.0 ms to 406.4 ms [16]. As calcium levels were not measured in the PROBeX study, we simulated calcium concentrations from a lognormal distribution based on the data used to build the original QTcF model [5]. Each patient was included 1000 times in the simulation data set to generate a large population and make the simulations less prone to randomness. The pharmacokinetic model was used to simulate M2 concentrations over the complete bedaquiline treatment period. We then used these M2 concentrations to simulate ECG measurements with the QTcF model for each virtual patient before starting bedaquiline and every week during treatment. QTcF values were simulated with and without the stochastic measurement error. Simulations were performed with and without concomitant clofazimine and assuming that no one received moxifloxacin per standard of care.

### Evaluation of ECG Monitoring Strategies

We initially explored sparse and intensive ECG monitoring strategies. The sparse strategy had ECG monitoring before starting bedaquiline and at weeks 2 and 12 after treatment start. With the intensive strategy, routine ECG monitoring took place before starting bedaquiline, at week 2, and every month from week 4 after treatment start. These 2 strategies were used as a starting point from which we explored similar monitoring schedules to identify the most suitable ECG monitoring strategy. The aim of an ECG monitoring strategy is to identify the patients who need to interrupt treatment for safety while minimizing identification of patients who can safely continue treatment. To evaluate each monitoring strategy, we determined the proportion of virtual patients missed who needed to interrupt treatment and the proportion of virtual patients who interrupted treatment but could have continued.

**Figure 1.**
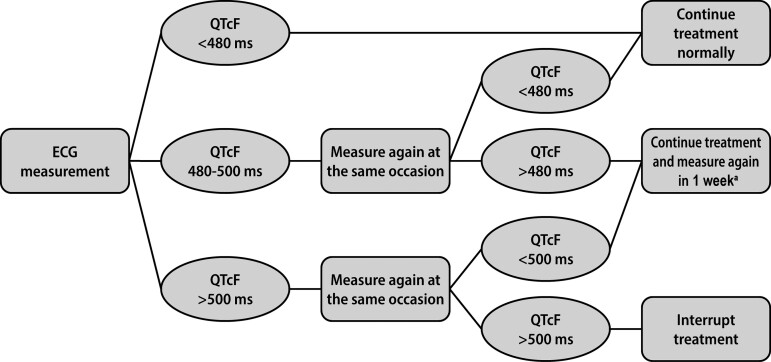
Flowchart of the decision algorithm for QTcF-interval prolongation during bedaquiline treatment. The algorithm was based on the 2014 World Health Organization guidelines [6]. ^a^Maximum twice following each planned monitoring occasion. Abbreviation: QTcF, Fridericia-corrected QT.

The simulated QTcF with measurement error was used for treatment decisions. The decision to continue or interrupt bedaquiline treatment of virtual patients was determined by an algorithm depicted in the flowchart in Figure 1. This algorithm was based on the 2014 WHO guidelines [6]. A prolonged QTcF interval >480 ms was confirmed by a second ECG measurement at the same occasion after the patient was allowed to rest. If both the first and second QTcF measured >500 ms, interruption of bedaquiline treatment was warranted. If the second ECG did not warrant interrupting bedaquiline treatment but the QTcF was still >480 ms, an extra ECG was performed after 1 week. A prolonged QTcF interval was monitored weekly until stable, which was defined by no indication to interrupt treatment following 2 weeks of extra ECG monitoring.

**Figure 2.**
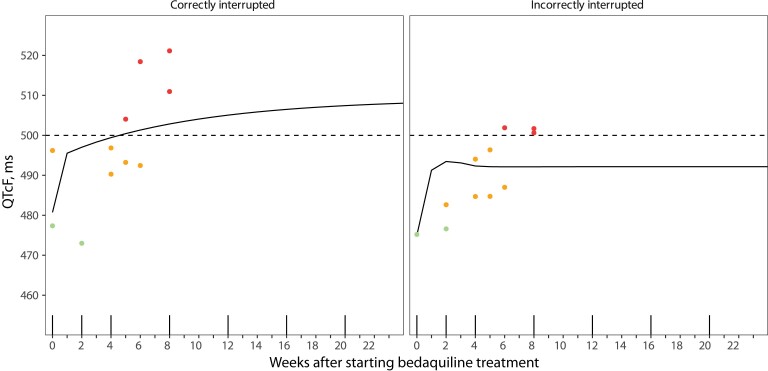
Example of virtual patients who correctly and incorrectly interrupted bedaquiline treatment following the treatment decision algorithm and intensive ECG monitoring strategy. The vertical solid lines on the x-axis indicate when routine ECG monitoring takes place with this strategy. The dots represent simulated QTcF with measurement errors, and the solid lines represent the simulated QTcF without measurement errors, that is, the true QTcF. QTcF measurements <480 ms are depicted by green dots, QTcF 480–500 ms by orange dots, and QTcF measurements >500 ms (horizontal dashed line) by red dots. Both virtual patients interrupted bedaquiline treatment after 2 consecutive QTcF measurements >500 ms at week 8. As we define a patient needing to interrupt treatment by having a true QTcF >500 ms at any point during treatment, we regard interrupting treatment as the correct decision for the first patient and as the incorrect decision for the second patient. Abbreviations: ECG, electrocardiogram; QTcF, Fridericia-corrected QT.

Whereas the simulated QTcF *with* measurement error was used to determine if a patient interrupted treatment, the simulated QTcF *without* measurement error was used to determine if a virtual patient correctly interrupted bedaquiline treatment or not. A virtual patient truly needing to interrupt treatment was defined by having a QTcF without measurement error >500 ms at any of the weekly simulated measurements during bedaquiline treatment. A virtual patient who did not need to interrupt treatment following this definition but who was nevertheless determined to need to interrupt treatment according to the treatment decision algorithm was considered to have interrupted treatment incorrectly. This is illustrated in an example of patients who correctly and incorrectly interrupted bedaquiline treatment in Figure 2.

### Software and Modeling Methodology

Model development was performed using NONMEM (version 7.4) with Pirana as the graphical interface [17, 18]. PsN, version 4.8, was used to automate multistep procedures in NONMEM [18]. R, version 3.6.3, was used for data management, data visualization, and statistics [19]. The Xpose4 R package, version 4.7, was used for graphical visualization of the visual predictive checks (VPCs) [18]. The VPCs were performed with n = 1000 simulated replicates of the original data set design.

In NONMEM, the first-order conditional estimation method with interaction was used for estimation of the pharmacodynamic parameters. Goodness-of-fit plots were used to evaluate the performance of the model. Parameter uncertainty was obtained from the Hessian covariance matrix as determined by NONMEM. The NONMEM PRIOR subroutine was used to incorporate prior information for all model parameters except the stochastic residual errors [15]. The priors of fixed parameters were weighted by the full variance–covariance matrix and assumed to be normally distributed. Prior information of random parameters was weighted by degrees of freedom and was assumed to be inverse-Wishart distributed.

## RESULTS

### Data

Study participants were included in the modeling analysis if they had available plasma concentrations for bedaquiline and M2, the known day that bedaquiline treatment was started, and ECG results. One hundred seventy out of 195 participants met these criteria and were included in the current analysis. The most common reason for excluding a participant was missing PK data. The characteristics of the participants included in the current analysis are shown in Table 1. Fewer observations were available at later months after starting treatment. We removed the only 2 samples in which both bedaquiline and M2 were below the limit of quantification. The majority of participants were on clofazimine during the complete treatment period. Approximately one-quarter of the participants received moxifloxacin at 1 of their included ECG measurement times (received ≤24 hours from the ECG); this was only at ECG measurements before starting bedaquiline treatment in all cases.

**Table 1.** Summary of Participant Characteristics

		All Participants	Sparse Sampling	Intensive PK Substudy
Patients included in the analysis, No.		170	150	20
Plasma PK observations, No.		1131	756	375
ECG measurements, No.		1702	1519	183
Age, median (IQR), y		33 (28–41)	33 (28–41)	31 (27–46)
Female sex, No. (%)		92 (54)	81 (54)	11 (55)
Race	Black, No. (%)	140 (82)	135 (90)	5 (25)
	Mixed, No. (%)	28 (16)	13 (8.7)	15 (75)
	White, No. (%)	2 (1)	2 (1.3)	0 (0)
Total body weight at baseline, median (IQR), kg		56 (49–63)	55 (49–61)	56 (46–67)
Albumin at baseline, median (IQR), g/L		3.4 (2.9–3.9)	3.3 (2.8–3.7)	3.8 (3.5–3.9)
Potassium at baseline, median (IQR), mmol/L		4.3 (4.0–4.7)	4.2 (4.0–4.7)	4.5 (4.2–4.7)
Patients receiving concomitant QTcF-prolonging drugs^a^	Clofazimine, No. (%)	167 (98.2)	148 (98.7)	19 (95)
	Moxifloxacin, No. (%)	44 (25.9)	44 (29.3)	0 (0)
HIV positive, No. (%)		105 (62)	96 (64)	9 (45)
Antiretroviral therapy	LPVr-based, No. (% of HIV+)	18 (17)	14 (15)	4 (44)
	Nevirapine-based, No. (% of HIV+)	87 (83)	82 (85)	5 (56)

Abbreviations: ECG, electrocardiogram; IQR, interquartile range; LPVr, lopinavir-ritonavir; PK, pharmacokinetic.

a Received ≤24 hours from any included study ECG time point.

### Pharmacokinetic/Pharmacodynamic Modeling

**Figure 3.**
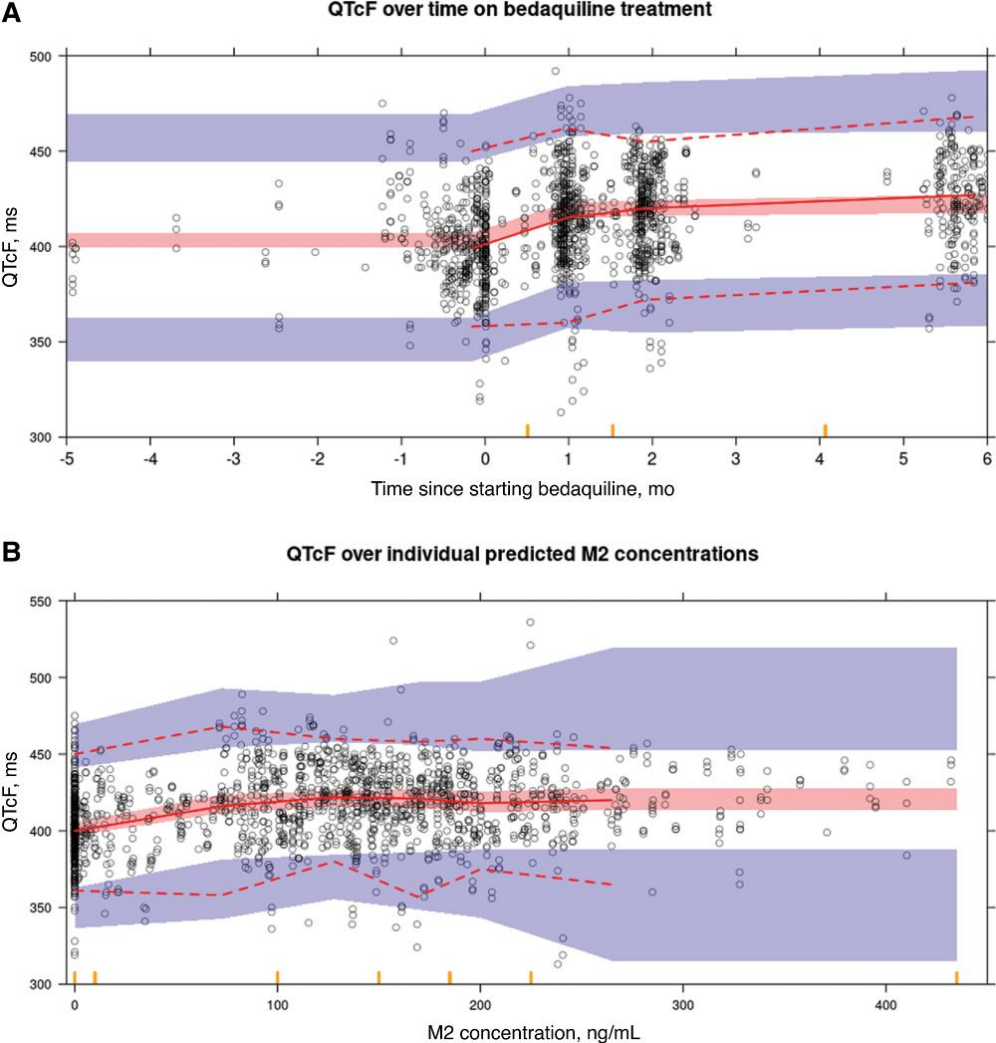
Visual predictive checks of the final QTcF model. The solid lines represent the 50th percentile of the observed QTcF times, and the dashed lines represent the 2.5th and 97.5th percentiles. The observed data before initiating and during bedaquiline treatment are depicted by open circles. The shaded areas represent the simulation-based 95% confidence intervals of the 2.5th, 50th, and 97.5th percentiles. The orange tick marks on the x-axes represent the boundaries of the bins used in the generation of the visual predictive checks. Abbreviation: QTcF, Fridericia-corrected QT.

While the PROBeX participants had lower M2 concentrations than the participants from the original modeling work, the PK model fitted the data well and individual model-predicted M2 plasma concentrations corresponded well with observed M2 concentrations (Supplementary Figure 1). The predicted median M2 concentration at ECG time points for PROBeX participants not receiving LPVr treatment was 142 ng/mL compared with 224 ng/mL in the original modeling work [5]. Final model parameter estimates for the QTcF model are shown in Table 2, and VPCs of the final model are shown in Figure 3.

**Table 2.** Final QTcF Model Parameters, Priors, and Their Uncertainty

Submodel	Parameter	Estimated Value (RSE%)	IIV CV% (RSE%)	Prior Value (RSE%)	Prior IIV CV% (RSE%)
Baseline	QTcF_0_, ms	400 (0.6)	3.8 (5.3)	400 (0.328)	3.75 (3.80)
M2 effect	E_max_, ms	28.5 (14.8)	–	28.6 (13.6)	–
	EC_50_, ng/mL	844 (29)	149.3 (7.1)	855 (24.4)	148 (11.8)
Time effect	QT_max_, ms	7.6 (9.3)	167.9^a^ (6.4)	6.50 (11.8)	167^a^ (12.7)
	T_1/2_, wk	6.87 (13.9)	–	6.44 (17.9)	–
Circadian rhythm effect	Amplitude_24_, ms	3.05 (51.5)	–	2.76 (43.9)	–
	Acrophase_24_, h	4.61 (42.7)	–	4.91 (26.6)	–
	Amplitude_12_, ms	1.71 (24.7)	–	1.46 (26.7)	–
	Acrophase_12_, h	4.29 (29.6)	–	4.50 (23.4)	–
Comedication^b^	Clofazimine, ms	11.4 (11.5)	–	11.8 (15.6)	–
	Moxifloxacin, ms	3.06 (63.7)	–	2.47 (98.4)	–
Covariate effects on the baseline QTcF	Calcium, ms per mmol/L^c^	−8.74 FIXED	–	−8.74 (28.3)	–
	Potassium, ms per mmol/L^c^	−1.49 (29.5)	–	−1.25 (38.5)	–
	Female, ms	7.16 (18.2)	–	7.75 (19.1)	–
	Black race, ms	−5.2 (26)	–	−6.86 (21.3)	–
	Age, ms per year^c^	0.388 (13.9)	–	0.349 (17.0)	–
Residual errors	Additive error, ms^d^	12.3 (7)	18.5 (11.4)	8.19 (1.81)	21.2 (11.2)
	Box-Cox IIV additive error	4.82 (15.9)	–	4.11 (24.0)	–
	Additive replicate error, ms^d^	5.9 (4.4)	46 (8.5)	6.87 (1.47)	23.9 (5.57)
	Box-Cox IIV replicate error	1.09 (20.1)	–	0.825 (40.5)	–

Abbreviations: CV, coefficient of variation; EC_50_, M2 concentration at which half of the maximum QTcF prolongation is reached; E_max_, maximum increase in QTcF by M2; IIV, interindividual variability; QTcF, Fridericia-corrected QT; QT_max_, maximum time effect on QTcF; RSE, relative standard error; T_1/2_, time at which half of the maximum time effect on QTcF is reached.

a The IIV in QT_max_ of the time effect was coded with a proportional model as opposed to an exponential model.

b Received ≤24 hours from any included study ECG time point.

c Implemented as ms per unit of deviation from the median population value.

d No prior information was incorporated for the estimation of residual errors.

**Figure 4.**
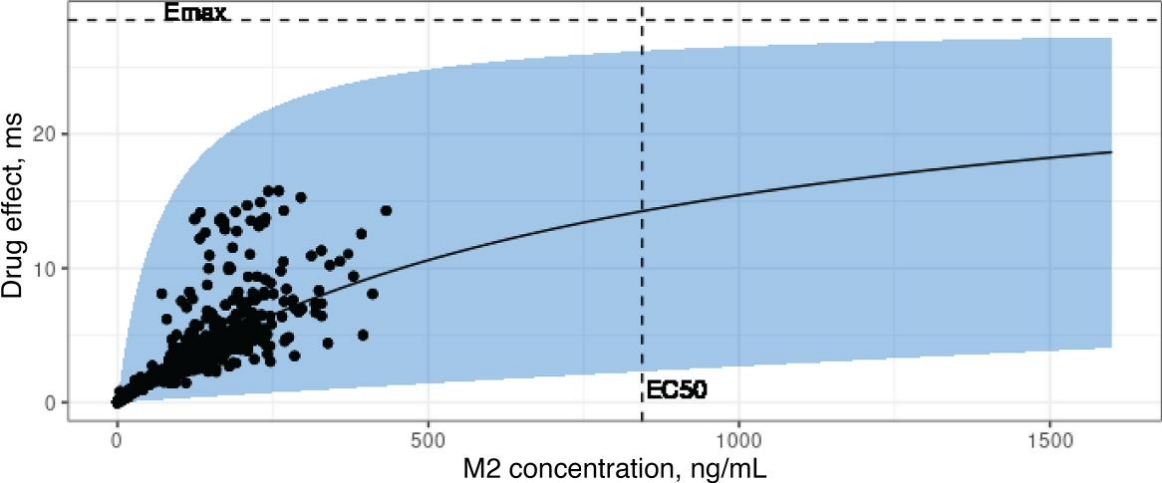
Predicted effect size of M2 on QTcF time. The black line represents the typical drug effect, and the blue band represents the 90% prediction interval resulting from the interindividual variability in EC_50_. The dots show the model-predicted drug effect at the times of ECG measurements, and the dashed lines indicate the E_max_ and EC_50_ of 28.5 ms and 844 ng/mL, respectively. The prediction interval was determined using the theoretical distribution of the interindividual variability in EC_50_. Abbreviations: EC_50_, M2 concentration at which half of the maximum QTcF prolongation is reached; ECG, electrocardiogram; QTcF, Fridericia-corrected QT.

The E_max_ concentration–response relationship between M2 and QTcF-interval prolongation and its IIV is shown in Figure 4. The IIV in EC_50_ was estimated to be very large (288% coefficient of variation). Individual model-predicted M2 plasma concentrations were all well below the estimated typical EC_50_.

### ECG Monitoring Simulations

**Figure 5.**
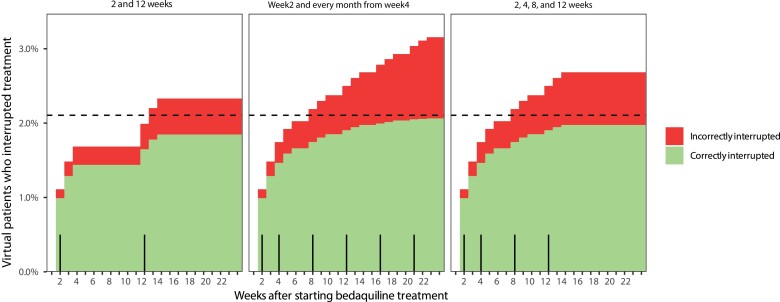
Cumulative percentage of virtual patients on bedaquiline treatment with concomitant clofazimine that interrupted treatment. From left to right, the graphs are for the sparse, intensive, and our proposed preferable ECG monitoring strategies. The horizontal dashed lines represent the total percentage of virtual patients who truly needed to interrupt bedaquiline treatment due to QTcF >500 ms at any point during treatment. The vertical solid lines on the x-axis indicate when routine ECG monitoring takes place. Abbreviations: ECG, electrocardiogram; QTcF, Fridericia-corrected QT.

Characteristics from 170 PROBeX participants were used to generate 170 000 virtual patients for the simulations. In the simulation including concomitant clofazimine, 2.1% of all virtual patients should really interrupt bedaquiline treatment at some point based on their QTcF. In the simulation without concomitant clofazimine, this was 0.7%. Table 3 shows the proportion of virtual patients who should interrupt bedaquiline treatment due to QTcF-interval prolongation but who were missed by the monitoring strategies, the proportion of virtual patients who interrupted bedaquiline treatment unnecessarily, and the average number of ECG monitoring occasions per virtual patient for each monitoring strategy in simulations with and without concomitant clofazimine. Both the sparse and intensive monitoring strategies were able to identify the majority of patients who should interrupt bedaquiline treatment due to QTcF-interval prolongation. The proportion of patients correctly identified with QTcF-interval prolongation >500 ms was higher in virtual patients treated with concomitant clofazimine compared with patients without concomitant clofazimine. More intensive monitoring identified more virtual patients who would interrupt treatment, both correctly and incorrectly. For the simulation with concomitant clofazimine, the weekly cumulative percentage of virtual patients who correctly and incorrectly interrupted treatment is shown over time in Figure 5 (shown in Supplementary Figure 2 for the simulation without concomitant clofazimine). With each additional ECG monitoring occasion, an increasing proportion of virtual patients who interrupted bedaquiline treatment could actually have continued treatment based on our criteria. With the intensive monitoring strategy, almost all virtual patients with QTcF >500 ms were identified by week 12, and later monitoring occasions predominantly falsely identified patients who would interrupt treatment. Monitoring fairly intensively, but only until 12 weeks (before treatment and at weeks 2, 4, 8, and 12), would be a preferable strategy. With this strategy, more virtual patients with QTcF-interval prolongation >500 ms were identified than with the sparse monitoring strategy. In addition, both the proportion of virtual patients falsely identified to interrupt treatment and the number of performed ECG monitoring occasions were significantly decreased compared with the full intensive monitoring strategy.

**Table 3.** Predicted Performance of ECG Monitoring Strategies for Patients on Bedaquiline Treatment With and Without Concomitant Clofazimine

Treatment	Strategy	Weeks When Monitoring Takes Place^a^	Patients With QTcF >500 ms Not Identified to Interrupt Treatment, %	Patients Incorrectly Identified to Interrupt Treatment, %	Average No. of ECG Monitoring Occasions per Patient
Bedaquiline	Sparse	0, 2, 12	15.5	26.2	3
	Intensive	0, 2, 4, 8, 12, 16, 20	2.7	42.6	7
	Preferred	0, 2, 4, 8, 12	7.9	32.2	5
Bedaquiline with concomitant clofazimine	Sparse	0, 2, 12	12.3	20.8	3
	Intensive	0, 2, 4, 8, 12, 16, 20	2.0	34.6	7
	Preferred	0, 2, 4, 8, 12	6.2	26.4	5

Abbreviations: ECG, electrocardiogram; QTcF, QTcF, Fridericia-corrected QT.

a Monitoring at week 0 takes place before bedaquiline treatment is initiated.

## DISCUSSION

In this work, we describe the relationship between concentrations of bedaquiline’s M2 metabolite and QTcF-interval prolongation in the South African PROBeX study. We provide insight on the performance of different ECG monitoring strategies through model-based simulations; this allows clinicians to make more informed decisions on how to monitor QTcF-interval prolongation during 6-month treatment with bedaquiline.

We predict that intensive ECG monitoring strategies identify the large majority of patients who should interrupt bedaquiline treatment due to QTcF >500 ms. However, with increasing intensity of ECG monitoring, it is also predicted that the proportion of patients with incorrectly interrupted treatment increases. In other words, as the prevalence of QTcF-interval prolongation in the treated population decreases over time, there is an increased chance that measurement errors will lead to falsely identifying patients who need to interrupt treatment. If ECG monitoring is performed monthly, most patients with QTcF >500 ms will be identified before 3 or 4 months of treatment duration, and subsequent monitoring occasions will predominantly falsely identify patients to interrupt treatment (ie, “false positives”).

It is important to weigh the risk of missing patients with dangerous QTcF-interval prolongation against the harm done when interrupting bedaquiline treatment for patients with multidrug-resistant tuberculosis unnecessarily. This consideration is complicated by the arbitrary nature of the cutoff used to guide interruption of treatment (QTcF >500 ms). As there is no established threshold effect for the relationship between QTcF and risk of life-threatening cardiac events, the risk associated with a QTcF slightly below 500 ms may not be much smaller than the risk associated with a QTcF slightly above 500 ms [20, 21]. Most virtual patients who were specified as having incorrectly interrupted treatment still had a QTcF close to 500 ms, as shown in Supplementary Figure 3. If only the risk associated with QTcF is considered, it may be argued that for many of these patients the decision to interrupt bedaquiline treatment was correct after all. However, the substantial mortality rate and limited treatment options for drug-resistant tuberculosis patients should be considered carefully in the decision to interrupt treatment due to QTcF-interval prolongation.

Current WHO guidelines recommend more intensive ECG monitoring if bedaquiline and LPVr are co-administered [8]. The rationale is that exposure to bedaquiline is increased by LPVr, which was thought to result in an increased risk of QTcF-interval prolongation. While LPVr inhibits the clearance of both bedaquiline and M2, it was previously shown that achieved exposure to M2 during the 24-week treatment period is actually lowered as it is formed through the clearance of bedaquiline and accumulation of M2 is slower when bedaquiline is cleared more slowly [14]. Because M2 drives the QTcF-interval prolongation, the risk of prolongation with concomitant LPVr is actually decreased, and we believe that more intensive monitoring is not necessary.

It is extremely difficult, if not impossible, to simulate treatment decisions made by clinicians. Instead, treatment decisions for virtual patients were made with an easy-to-use algorithm in an effort to approximate treatment decisions by a real clinician. In clinical practice, treatment decisions may be made that differ from our algorithm and that could result in different outcomes. Notably, we conducted the ECG monitoring simulations without including parameter uncertainty, and sensitivity to changes in parameter values was not explored.

For translation of these results to other populations, demographic differences and concomitant drugs affecting the prevalence of QTcF-interval prolongation should be taken into account. The comparison between the simulations with and without concomitant clofazimine showed that it is harder to correctly identify QTcF >500 ms when the prevalence is lower. However, the results are otherwise comparable, such that the same monitoring recommendation can be made for patients on bedaquiline treatment with and without concomitant clofazimine. While not explored in these simulations, the same can be expected for other concomitant drugs that prolong the QTcF interval.

The modeling in this analysis is supported by prior information from a previously established QTcF model developed on data from 2 phase II studies [5]. These data were found to differ from the PROBeX data we used: Individual M2 plasma concentrations at the time points of ECG measurements were lower in our model-based predictions compared with those for the 2 phase II studies (median, 142 ng/mL vs 224 ng/mL). These studies performed ECG monitoring at week 2 in addition to months 1, 2, and 6, like in the PROBeX study. As M2 plasma concentrations are known to be highest following the loading-dose phase just after week 2 [13], the lack of an extra ECG at week 2 may be one explanation for predicting lower concentrations in PROBeX. The difference in race proportions between PROBeX and the phase II studies could be another explanation. Black race was much more prevalent in the PROBeX study compared with the phase II studies (82% vs 36%). Black race has been associated with increased clearance of bedaquiline and M2 and lower concentrations of both as a result [13, 22, 23].

The final QTcF model parameter estimates were comparable to the prior values, and their precision was mostly similar (Table 2) [5]. However, the estimate of the effect of concomitant moxifloxacin on QTcF was associated with a higher precision than in the prior information. Furthermore, during model development, we detected a slight underprediction of QTcF by the model at later months during the treatment. This could be due to the accumulation of clofazimine over time. Many of the PROBeX participants started clofazimine at the same time as bedaquiline, and clofazimine is known to accumulate slowly with the standard dosing regimen [24]. Although clofazimine was included in the model as a covariate, its accumulation over time was not accounted for. This may also explain why we predict a smaller-than-expected effect of concomitant clofazimine use on QTcF [25]. Estimation of a time-varying effect of clofazimine on QTcF was not an option as the PROBeX data did not allow its unique identification from the effect of time on treatment, which was already incorporated in the model.

## CONCLUSIONS

We predict that both sparse and intensive ECG monitoring strategies identify the majority of patients who should interrupt bedaquiline treatment due to a QTcF interval >500 ms. Weighing the risk of missing patients with substantial QTcF-interval prolongation and the harm of interrupting bedaquiline treatment unnecessarily is important but not easy to do. We propose monitoring the ECG before treatment and at weeks 2, 4, 8, and 12 after starting 6-month bedaquiline treatment. For this strategy, we recommend not performing routine ECG monitoring after week 12 as that is likely to lead to unnecessary treatment interruptions. In contrast to current WHO guidelines, we advise that increased intensity of ECG monitoring is not needed if bedaquiline is co-administered with LPVr.

## Supplementary Material

ofac372_Supplementary_Data

## Supplementary Data

Supplementary materials are available at *Open Forum Infectious Diseases* online. Consisting of data provided by the authors to benefit the reader, the posted materials are not copyedited and are the sole responsibility of the authors, so questions or comments should be addressed to the corresponding author.
