# Supplementary material for: Model-Predicted Impact of ECG Monitoring Strategies During Bedaquiline Treatment
Source: Open Forum Infect Dis. 2022 Jul 27;9(8):ofac372. doi: 10.1093/ofid/ofac372 (PMC9420883; doi:10.1093/ofid/ofac372)
Supplement: ofac372_Supplementary_Data [file ofac372_supplementary_data.docx]

**Supplementary material to: Model-predicted impact of ECG monitoring strategies during bedaquiline treatment**

Authors: Stijn W. van Beek^1^, Lénaïg Tanneau^2^, Graeme Meintjes^3^, Sean Wasserman^3,4^, Neel R. Gandhi^5,6^, Angie Campbell^5^, Charle A. Viljoen^7,8^, Lubbe Wiesner^9^, Rob Aarnoutse^1^, Gary Maartens^3,9^, James C.M. Brust^10^*, Elin M. Svensson^1,2^*
*Authors contributed equally to the manuscript

Affiliations:

1 Department of Pharmacy, Radboud Institute for Health Sciences, Radboud University Medical Center, Nijmegen, the Netherlands

2 Department of Pharmacy, Uppsala University, Uppsala, Sweden

3 Wellcome Centre for Infectious Diseases Research in Africa, Institute of Infectious Disease and Molecular Medicine, Department of Medicine, University of Cape Town, Cape Town, South Africa

4 Division of Infectious Diseases and HIV Medicine, Department of Medicine, University of Cape Town, South Africa

5 Departments of Epidemiology & Global Health, Rollins School of Public Health, Emory University, Atlanta, Georgia, USA

6 Division of Infectious Diseases, Department of Medicine, Emory School of Medicine, Emory University, Atlanta, Georgia, USA

7 Division of Cardiology, Department of Medicine, University of Cape Town, Cape Town, South Africa

8 Cape Heart Institute, Faculty of Health Sciences, University of Cape Town, Cape Town, South Africa

9 Division of Clinical Pharmacology, Department of Medicine, University of Cape Town, South Africa

10 Division of General Internal Medicine, Department of Medicine, Albert Einstein College of Medicine, Bronx, NY, USA

**Supplementary Figure 1.** Pharmacokinetic goodness-of-fit plot of individual model-predicted M2 plasma concentrations versus observed M2 plasma concentrations. The black line represents the line of unity and the blue line a smooth fitting the data.


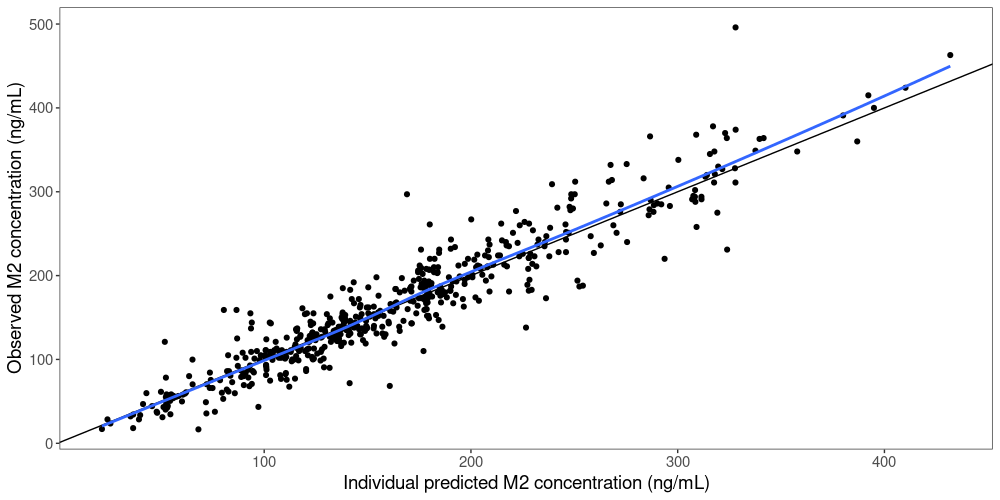

**Supplementary Figure 2.** Cumulative percentage of virtual patients on bedaquiline treatment without concomitant clofazimine that interrupted treatment. From left to right, the graphs are for the sparse, the intensive and our proposed preferable ECG monitoring strategies. The horizontal dashed lines represent the total percentage of virtual patients that truly needed to interrupt bedaquiline treatment due to QTcF >500 ms at any point during treatment. The vertical solid lines on the x-axis indicate when routine ECG monitoring takes place.

**Supplementary Figure 3.** Predicted distribution of maximum simulated QTcF without stochastic measurement error during treatment of virtual patients receiving bedaquiline with (**A**) and without (**B**) concomitant clofazimine who interrupted treatment following the ECG monitoring strategy measuring before treatment, at week 2 and every month from week 4. In red are the patients who incorrectly interrupted treatment (QTcF <500 ms) and in green the patients who correctly interrupted treatment (QTcF >500 ms).
